# Target and Non-Target Approaches for Food Authenticity and Traceability

**DOI:** 10.3390/foods10010172

**Published:** 2021-01-16

**Authors:** Joana S. Amaral

**Affiliations:** 1 Centro de Investigação de Montanha (CIMO), Instituto Politécnico de Bragança, Campus de Sta. Apolónia, 5301-857 Bragança, Portugal; jamaral@ipb.pt; Tel.: +351-273-383-138; 2 REQUIMTE-LAQV, Faculdade de Farmácia, Universidade do Porto, Rua de Jorge Viterbo Ferreira, 228, 4050-313 Porto, Portugal

In the last decade, consumers have become increasingly aware of and concerned about the quality and safety of food, in part due to several scandals that were widely disseminated by the media. Currently, consumers are requesting more information about the food they buy, not only from a nutritional point of view but also regarding origin, safety, traceability, and authenticity. In addition, concerns about environmental and ethical issues are on the rise, with more attention being given to topics such as biodiversity protection, production mode, and food authenticity. The growing demand for higher quality foods, the desire for new experiences associated with delicacy products or foods having particular organoleptic characteristics, together with the increasing willingness to pay more money for such products, provides an overall incentive for the adulteration of premium foods. Moreover, several factors such as international trade, market globalization, long and complex food supply chains, and the booming of e-commerce, further create opportunities for food fraud. While in several cases food adulteration poses no major risk for consumers’ health (e.g., mislabeling of geographical origin), in others it can result in health hazards due to toxic or allergenic substances. However, even when health is not jeopardized, food fraud leads to unfair market competition and consumers being deceived. For all these reasons, the issue of food authenticity and food fraud has been receiving increased attention from several stakeholders, including government agencies and policymakers, control labs, producers, industry, and the research community, and different attempts have been made aiming for the definition of these concepts. According to the CEN Workshop Agreement 17369:2019, an authentic food product is “*a food product where there is a match between the actual food product characteristics and the corresponding food product claims; when the food product actually is what the claim says that is*” [1,2]. In the discussion paper on food integrity and food authenticity of the working group of the Codex Alimentarius Commission [3], food fraud is described as “*any deliberate action of businesses or individuals to deceive others in regards to the integrity of food to gain undue advantage*”. Moreover, four key elements are identified, namely deliberate intent, deception, financial gain and misrepresentation, which are in line with the European Commission’s key criteria to refer to when establishing if a case should be considered as fraud or as non-compliance, namely (i) violation of one or more rules of the European Union agri-food chain legislation as referred to in Article 1(2) of Regulation (EU) 2017/625, (ii) customer deception, (iii) economic gain, (iv) intention [2,4]. Furthermore, different types of food fraud have been described, including substitution, dilution, mislabeling, concealment, and unapproved enhancement, among others [2]. In order to identify, tackle and/or deter fraudulent practices in the agri-food sector, complementary approaches are needed to address this complex issue, including analytical testing and broader strategies such as implementing early warning systems, vulnerability assessments, and intelligence gathering, among which the development of new, fast and advanced analytical methods for checking food authenticity is a central aspect. Thus, several works have been published on the subject with respect to different food matrices, putting in evidence a variety of analytical techniques that can be used for food authentication [2,5,6,7,8,9,10]. So far, the majority are targeted methods, which look for a pre-defined characteristic or adulterant, thus being focused on the detection of a few selected analytes [11,12,13]. However, in the last few years, non-targeted methods have increasingly come into focus. These methods do not rely on the analyses of selected individual analytes since the molecules to be detected are not known a priori, but instead aim at studying a global fingerprint that should be as comprehensive as possible [11,12,13]. This approach can be advantageous when no information about possible adulterants is yet known and/or when unconventional adulterants are added, which would be unlikely to be detected by conventional targeted approaches. Moreover, contrary to targeted methods that frequently need complex and expensive extraction processes, in non-targeted approaches a simple sample preparation is generally performed to get as many matrix components as possible [12]. Despite the many challenges that still need to be overcome, non-targeted methods are becoming increasingly used and their contribution to deterring food fraud, together with targeted methods, is expected to grow in the coming years.

In this regard, this Special Issue aimed at gathering original research and review papers focusing on the development and application of both targeted and non-targeted methodologies to verify food authenticity and traceability. This Special Issue includes eighteen notable contributions, comprising one review paper and seventeen original research papers, these last dealing with the authentication of different foods, including some considered as highly prone to food fraud such as olive oil [14,15], honey [16,17], fish [18,19,20] and meat [21,22,23,24].

Several research articles in this Special Issue reported the application of different analytical techniques including chromatography, spectrometry, and spectroscopy aiming for food authentication. Grazina et al. [18] used a targeted approach to determine nineteen fatty acids by gas-chromatography with flame ionization detection (GC-FID), which were used together with advanced chemometrics to discriminate wild from farmed salmon. Based on seventeen features obtained from the chemical analysis, all the tested approaches, namely principal components analysis (PCA), *t*-distributed stochastic neighbor embedding (*t*-SNE), and seven machine learning classifiers, allowed them to differentiate the two groups (wild vs. farmed). Moreover, five classifiers allowed distinguishing between groups of farmed salmon from different geographical origins. Detecting mislabeling of geographical origin is an issue that has been receiving increasing attention in the last few years, since certified products or those produced in certain regions are frequently associated with a higher price due to their quality and specific characteristics. Analytical testing for identifying the geographical origin of foods is generally considered of high complexity since specifications for agri-food products with geographic indication are frequently based on subjective characteristics such as organoleptic properties [25]. Kim et al. [26] reported the use of hydrophilic and lipophilic metabolite profiling by gas chromatography-mass spectrometry (GC-MS) coupled with orthogonal partial least squares discriminant analysis (OPLS-DA) to differentiate perilla and sesame seeds originating from China and Korea. Furthermore, the authors noticed that glycolic acid was a notable metabolite for discriminating between perilla seeds grown in China and Korea and proposed this compound as being a potential biomarker for such discrimination. Likewise, proline and glycine could be considered potential biomarkers to determine the geographical origin of sesame seeds. The importance of tracing the geographical origin was also addressed in the study of Vukašinović-Pešić et al. [16] on multifloral honeys from different regions of Montenegro. The mineral content determined by inductively coupled plasma-optical emission spectrometry (ICP-OES) and linear discriminant analysis allowed the researchers to distinguish honeys that originated from areas exposed to industrial pollution. A different approach was proposed by Lippoli et al. [17] aiming for the fast authentication of honey’s geographical origin. The authors describe the development of a non-targeted method using direct analysis in real time and high resolution mass spectrometry (DART-HRMS) combined with multivariate statistical analysis to discriminate chestnut honey from Portugal and Italy and acacia honey from Italy and China. A non-targeted method coupled with chemometrics was also the approach selected by Barbieri et al. [14] towards the authentication of virgin olive oils. In this study, a classification model was developed based on the raw data from the volatile fraction fingerprint obtained by flash gas chromatography and partial least squares-discriminant analysis (PLS-DA) to predict the commercial category of olive oils (extra virgin, virgin and lampante). The proposed classification model was shown to be robust since it included a high number of diversified samples classified by sensorial analysis (*n* = 331); it was also shown to have good performance, since it was able to correctly classify a high percentage of samples in both cross and external validation. Thus, the proposed approach represents a valid alternative for supporting official sensory panels and increasing the efficiency and fastness of controls, since it could be used as a screening tool allowing for a fast pre-classification of olive oil quality grade, thus supporting the panels by prioritizing the samples or even reducing the number of samples requiring sensory analysis. The comparison of targeted and non-targeted approaches for detecting the adulteration of fresh turkey meat by the fraudulent addition of protein hydrolysates was reported by Wagner et al. [21]. Turkey breast muscles were treated with plant or animal protein hydrolysates (those being produced by enzymatic and acidic hydrolysis and presenting different hydrolyzation degrees—partial or total) and analyzed by traditional high-performance liquid chromatography with ultraviolet-visible detection (HPLC-UV/VIS) targeting ten proteinogenic amino acids and by GC–MS and nuclear magnetic resonance (NMR) spectroscopy as two non-targeted metabolite profiling methodologies. While free amino acids analysis allowed the detection of the injection with fully hydrolyzed proteins, it was not suitable for the detection of food fraud in the case of partial hydrolysates. It was concluded that for lower hydrolyzation degrees, additional compounds originating from protein (such as sugars and the by-products released during hydrolysis) play an important role in the differentiation of nontreated samples and hydrolysate treated ones. Thus, the combination of amino acid profiling and additional compounds can provide stronger evidence for detecting and classifying this kind of adulteration. 

The feasibility of using spectroscopic techniques as non-targeted approaches for food authentication was also demonstrated in this Special Issue. Truffles are very expensive mushrooms whose price depends mainly on their species but also on their origin, with the white Piedmont truffle (*Tuber magnatum*) and the black Périgord truffle (*Tuber melanosporum*) being the most valued species. In the paper of Segelke et al. [27] Fourier transform near-infrared (FT-NIR) spectroscopy combined with chemometrics is used to differentiate these truffle species from other species that are less valued but morphologically very similar. Various data pre-processing techniques were evaluated to avoid overfitting and the results compared using several classification models. The results showed the ability to differentiate the expensive white truffle *T. magnatum* from *Tuber borchii* with 100% accuracy, and *T. melanosporum* from *Tuber aestivum* and some species of Chinese black truffles with an accuracy of 99%. Moreover, Piedmont truffles could be differentiated from non-Italian *T. magnatum* truffles with an accuracy of 83%. Therefore, this work demonstrates the potential of FT-NIR spectroscopy as a fast and low-cost authentication tool, not requiring special training for sample preparation and equipment handling, thus being very suited for the industrial screening of samples. 

In addition to chemical approaches, several works have been conducted so far describing the development and application of molecular biology techniques for food authentication purposes. These techniques are highly specific and sensitive and are frequently considered as the most suitable tools for the identification of species. Various research papers on the use of DNA-based approaches are also included in this Special Issue, from the comparison of different DNA extraction methods [28] to the use of multiplex polymerase chain reaction (PCR) [23], real-time PCR [19,22,24,29,30], or more advanced techniques such as Digital PCR [31]. Kim et al. [23] proposed the use of a simple qualitative assay based on the use of multiplex PCR to identify three deer species, namely red deer (*Cervus elaphus*), roe deer (*Capreolus capreolus*), and water deer (*Hydropotes inermis*). Three sets of species-specific primers were developed, generating amplicons of different sizes for each species that were then visualized by capillary electrophoresis to increase resolution and accuracy for the detection of the multiple targets. In other works, the specific identification of species was achieved by using real-time PCR. Kim et al. [24] designed new species-specific primers and probe targeting the *cytb* region of donkey (*Equus asinus*) allowing the detection of as low as 0.001% donkey meat in raw and processed meat mixtures made with beef. Velasco et al. [29] reported the development of a real-time PCR based on the use of specific primers and a minor groove binding TaqMan probe targeting the COI (*Cytochrome Oxidase I*) region for the specific authentication of common cuttlefish (*Sepia officinalis*) in seafood products. Commercial samples were also analyzed by FINS (forensically informative nucleotide sequencing) in order to test the reliability of the developed method and guarantee the correctness of the level of mislabeling found in this work (25%). This low-cost method proved to be reliable in the differentiation of this species from other cephalopods and can be very useful for food control authorities, since species from the genus *Sepia* are frequently similar and very difficult to identify after processing because the characteristics for morphological identification are eliminated. Kyriakopoulou and Kalogianni [15] described the development of a new allele-specific real-time PCR to specifically differentiate olive oil from the valuable wild-type *Olea europaea* var *Sylvestris* from the commonly cultivated type *Olea europaea* L. var *Europaea*. Besides being used for species-specific identification, real-time PCR is also reported for quantification purposes [22,29,30]. While Oh et al. [29] estimate the percentage of corn (*Zea mays*) as an added adulterant in turmeric powder (*Curcuma longa*) by using the fluorescent dye SYBRGreen, others propose the use of specific probes [22,30]. Dolch et al. [22] developed two multiplex real-time PCR assays using specific primers and probes, one for the detection and quantification of chicken (*Gallus gallus*), guinea fowl (*Numida meleagris*) and pheasant (*Phasianus colchicus*), and other for quail (*Coturnix japonica*) and turkey (*Meleagris gallopavo*). For each system, three different quantification methods were compared for estimating the relative meat content of these poultry species in meat mixtures. According to the authors, each method had its pros and cons, although the matrix-specific multiplication factors method was the one presenting more accepted recovery rates. By the contrary, in the work of Grazina et al. [30] the ∆Ct method was chosen to estimate the percentage of *Ginkgo biloba* in commercial herbal infusions. The proposed normalized real-time PCR system, which required the amplification of the specific target (*G. biloba* ITS1 region) using the novel primer set and TaqMan probe and a reference endogenous gene (nuclear 18S rRNA), exhibited high performance parameters and was successfully validated using blind mixtures. To assess the occurrence of fraud in the swordfish supply chain, Ferrito et al. [20] suggested the use of a different molecular strategy encompassing the PCR amplification of the frequently used barcode COI gene combined with the restriction fragment length polymorphism (RFLP) technique. The COIBar-RFLP procedure was applied on several authenticated reference samples of swordfish (*Xiphias gladius*) and four different shark species to generate species-specific restriction enzyme patterns. Those were further used for the authentication of fresh and frozen commercial swordfish slices, allowing the detection of *Prionace glauca*, *Mustelus mustelus* and *Oxynotus centrina* in slices labeled as *Xiphias gladius*. A different technology, namely digital PCR, is reported in the work of Morcia et al. [31] to identify economically motivated adulteration in the pasta industry by the substitution of *Triticum durum* with cheaper common wheat (*Triticum aestivum*). Moreover, the proposed assay allowed the researchers to track the adulterant down to 3%, which is the critical value established in the legislation as a limit for accidental contamination. 

Finally, closing this Special Issue, the review paper by Hassoun et al. [32] discusses the use of different analytical methods for detecting frauds in food products of animal origin, with particular attention being paid to non-targeted spectroscopic detection methods. The advantages, opportunities and challenges associated with the use of spectroscopic techniques are discussed and several application examples are given, covering relevant and recently published works. 

Overall, the papers included in the Special Issue “Target and Non-Target Approaches for Food Authenticity and Traceability” put in evidence the global relevance of the topic and the importance of developing different approaches that can be used by control laboratories and governmental agencies to verify and guarantee food authenticity and traceability, allowing agencies to detect and expose eventual food fraud scenarios, and therefore protecting producers and industry from unfair competition as well as increasing consumers’ confidence in purchased foods.
